# Supplementary material for: What are housekeeping genes?
Source: PLoS Comput Biol. 2022 Jul 13;18(7):e1010295. doi: 10.1371/journal.pcbi.1010295 (PMC9312424; doi:10.1371/journal.pcbi.1010295)
Supplement: S1 Text — Supplementary method in this text is method used for scoring whole-body C. elegans RNAi screens. Also shown are the supplementary figures, captions, and captions for the supplementary tables. (DOCX) [file pcbi.1010295.s001.docx]

Supplementary Text

# Supplementary results

## Definition of housekeeping genes lacks experimental basis

We scoured the literature from Google Scholar using Herzing’s Publish or Perish[1] software. From the top hits with highest citations among more than 109,000 search results, we looked at the text and title of articles containing the words: housekeeping, genes, maintenance, and required. Excerpts from the most highly cited articles have been listed in the Supplementary text. We found that none of these articles reference an article which constructs the experimental basis of the definition of housekeeping genes. This suggests that, given the pervasiveness and usefulness of the term, there is a need to provide an experimental basis for existence of housekeeping genes. The list of definitions used in some of the highly referenced papers is below:

1. Thellin, Zorzi, Lakaye, De Borman, Coumans, Hennen, Grisar, Igout, and Heinen, 1999 (Cited 1761) – “All these techniques can use internal standards, mainly housekeeping genes, so called because their synthesis occurs in all nucleated cell types since they are necessary for the cell survival.”[2]

2. Eisenberg and Levanon, Trends in Genetics, 2003 (Cited – 754) - “… most genes show constitutive expression in only a subset of tissues, some gene products are required for the maintenance of the basal cellular function and are constitutively found in all human cells. These genes are called housekeeping genes.”[3]

3. Eisenberg and Levanon, Trends in Genetics, 2013 (Cited – 726) – “Housekeeping genes are involved in basic cell maintenance and, therefore, are expected to maintain constant expression levels in all cells and conditions.” “Housekeeping genes are genes that are required for the maintenance of basal cellular functions that are essential for the existence of a cell, regardless of its specific role in the tissue or organism.”[4]

4. Warrington, Nair, Mahadevappa, and Tsyganskaya, Physiological Genomics, 2000 (Cited - 603) – “housekeeping genes, or maintenance genes, are those genes constitutively expressed to maintain cellular function.”[5]

5. Butte, Dzau, and Glueck, Physiological Genomics, 2001 (Cited – 212) – “Housekeeping genes are constitutively expressed to maintain cellular function.”[6]

6. Zhu, He, Hu, and Yu, Trends in Genetics, 2001 (Cited - 260) – “Housekeeping (HK) genes are ubiquitously expressed in all tissue and cell types and constitute the basal transcriptome for the maintenance of basic cellular functions.”[7]

While we did not find an experimental basis from literature search, we did find a commonality. The housekeeping genes appeared to delineate relationship between three types of biological features: (i) consistently or stably expressed genes, (ii) essential genes, and (iii) genes participating in cellular maintenance. In the subsequent sections, we will test multiple different datasets belonging to different organisms to establish relationship between these biological features.

## GAPDH may not be a good candidate as a housekeeping gene

Glyceraldehyde 3-phosphate dehydrogenase (GAPDH) is the most commonly used housekeeping gene to benchmark expression of other genes in qRT-PCR analyses. To define the appropriateness of GAPDH as a housekeeping gene, we started with previously published transcriptomics data belonging to CHO cells, hamster tissues[8], human tissues from Genotype-Tissue Expression (GTEx) project[9] and Human Protein Atlas (HPA)[10], and NCI-60 cancer cells (Klijn et al.[11], and CellMiner[12]). We calculated the G_C_ for these datasets and then compared the G_C_ of GAPDH across the 7 datasets.

Low G_C_ represents low variability in level of expression across tissues or samples, as would be expected for housekeeping genes. However, our analyses indicated that the G_C_ values for GAPDH were very different across all human and hamster datasets. For instance, Klijn et al. (i.e., NCI-60 cell lines) showed the lowest G_C_ value was at 14.6 percentile and hamster tissue had the highest G_C_ value at 57.6 percentile (Figure S1). The G_C_ values in human datasets varied by 18 percentiles while hamster and CHO data differed by 35.3 percentiles. Thus, the high variability in G_C_ percentiles indicate that GAPDH may not be a good candidate as a housekeeping gene as it is not as stably expressed as generally thought.

# Supplementary Methods

## Whole animal *C. elegans* RNAi screens

*C. elegans* rrf-3 mutants, which are hypersensitive to RNAi, were used. The details on how the RNAi worms were grown and prepared can be found in Ke et al. 2018[13]. Please see below for details on manual screening.

### Workflow for scoring

1. Open 1-2 L4440 representative reference images, leave them open in 2nd monitor.
2. Open well image to be scored.
3. Compare to reference images and decide whether size of worms is = or < than worms in reference image. Enter score in column “reduced size” (0 = WT, 1= reduced size).
4. Define whether smaller animals are gravids (small but have eggs) or larvae (small but no eggs): (i) Enter score in “gravids” column (1=gravids, 0=larvae), OR (ii) Enter score in "Larvae or egg less adult" column (0=gravids, 1=Larvae or egg less adult).
5. A second round of sorting was done based on the criterion described as follows: The sum of the scores from the column "reduced size" (which can be gravid with reduced size or larvae) was calculated. The maximum score could be 6 (3 repeats of the experiment, each scored independently by 2 personnel) or 4 if there were only 2 repeats of the experiment.
6. The genes were grouped based on the following criteria:

Group 1: High confidence hit

RNAi clone consistently leading to reduced size (it can be larvae or adult with reduced size).

For plates tested in triplicate: High confidence hits are those RNAi clones that scored 5 or 6 (out of 6 scores). This means that in all 3 repeats worms had reduced size (result was very reproducible).

For plates tested in duplicate: High confidence hits are those RNAi clones that scored 4. This means that the worms had reduced size in both the repeats.

Group 2: Medium confidence hit

For plates tested in triplicate: Medium confidence hits are those RNAi clones that scored 4. This means that in 2 repeats out of 3, worms had reduced size.

For plates tested in duplicate: Medium confidence hits are those RNAi clones that scored 3 (out of 4 scores).

Group 3: Wild Type BIOMASS

RNAi clones that scored 0-1 (in plates that were done in duplicate or triplicate).

Group 4: Untested

RNAi clones for which bacteria do not grow. Hence, worms in these wells are starved.

RNAi clones that did not grow in 2 out of 3 or 1 out of 2 trials,

Due to experimental issues, these clones cannot be called neither Wild Type nor reduced size. If these clones were critical to the model, they could be manually retested.

Group 5 Unknown

RNAi clones that have given variable results (scores 2-3), therefore we cannot say with confidence whether they are WT or show reduced size.

# References

1. Harzing AW. Publish or Perish. In: available from http://www.harzing.com/pop.htm [Internet]. 2007 [cited 15 Oct 2020]. Available: http://harzing.com/pop.htm

2. Thellin O, Zorzi W, Lakaye B, De Borman B, Coumans B, Hennen G, et al. Housekeeping genes as internal standards: Use and limits. J Biotechnol. 1999;75: 291–295. doi:10.1016/S0168-1656(99)00163-7

3. Eisenberg E, Levanon EY. Human housekeeping genes are compact. Trends Genet. 2003;19: 362–365. doi:10.1016/S0168-9525(03)00140-9

4. Eisenberg E, Levanon EY. Human housekeeping genes, revisited. Trends Genet. 2013;29: 569–574. doi:10.1016/j.tig.2013.05.010

5. Warrington JA, Nair A, Mahadevappa M, Tsyganskaya M. Comparison of human adult and fetal expression and identification of 535 housekeeping/maintenance genes. Physiol Genomics. 2000;2: 143–7. doi:10.1152/physiolgenomics.2000.2.3.143

6. Butte AJ, Dzau VJ, Glueck SB. Further defining housekeeping, or “maintenance,” genes Focus on “A compendium of gene expression in normal human tissues”. Physiol Genomics. 2001. doi:10.1007/s10857-005-4766-0

7. Zhu J, He F, Hu S, Yu J. On the nature of human housekeeping genes. Trends Genet. 2008;24: 481–484. doi:10.1016/J.TIG.2008.08.004

8. Shamie I, Duttke SH, Karottki KJLC, Han CZ, Hansen AH, Hefzi H, et al. A Chinese hamster transcription start site atlas that enables targeted editing of CHO cells. NAR genomics Bioinforma. 2021;3. doi:10.1093/NARGAB/LQAB061

9. GTEx Consortium TGte. The Genotype-Tissue Expression (GTEx) project. Nat Genet. 2013;45: 580–5. doi:10.1038/ng.2653

10. Uhlen M, Fagerberg L, Hallstrom BM, Lindskog C, Oksvold P, Mardinoglu A, et al. Tissue-based map of the human proteome. Science (80- ). 2015;347: 1260419–1260419. doi:10.1126/science.1260419

11. Klijn C, Durinck S, Stawiski EW, Haverty PM, Jiang Z, Liu H, et al. A comprehensive transcriptional portrait of human cancer cell lines. Nat Biotechnol. 2015;33: 306–312. doi:10.1038/nbt.3080

12. Shankavaram UT, Varma S, Kane D, Sunshine M, Chary KK, Reinhold WC, et al. CellMiner: A relational database and query tool for the NCI-60 cancer cell lines. BMC Genomics. 2009. doi:10.1186/1471-2164-10-277

13. Ke W, Drangowska-Way A, Katz D, Siller K, O’Rourke EJ. The ancient genetic networks of obesity: Whole-animal automated screening for conserved fat regulators. Methods in Molecular Biology. Humana Press Inc.; 2018. pp. 129–146. doi:10.1007/978-1-4939-7847-2_10

14. Xiong K, la Cour Karottki KJ, Hefzi H, Li S, Grav LM, Li S, et al. An optimized genome-wide, virus-free CRISPR screen for mammalian cells. Cell Reports Methods. 2021;1: 100062. doi:10.1016/J.CRMETH.2021.100062
