# Supplementary figures and images for: What are housekeeping genes?
Source: PLoS Comput Biol. 2022 Jul 13;18(7):e1010295. doi: 10.1371/journal.pcbi.1010295 (PMC9312424; doi:10.1371/journal.pcbi.1010295)

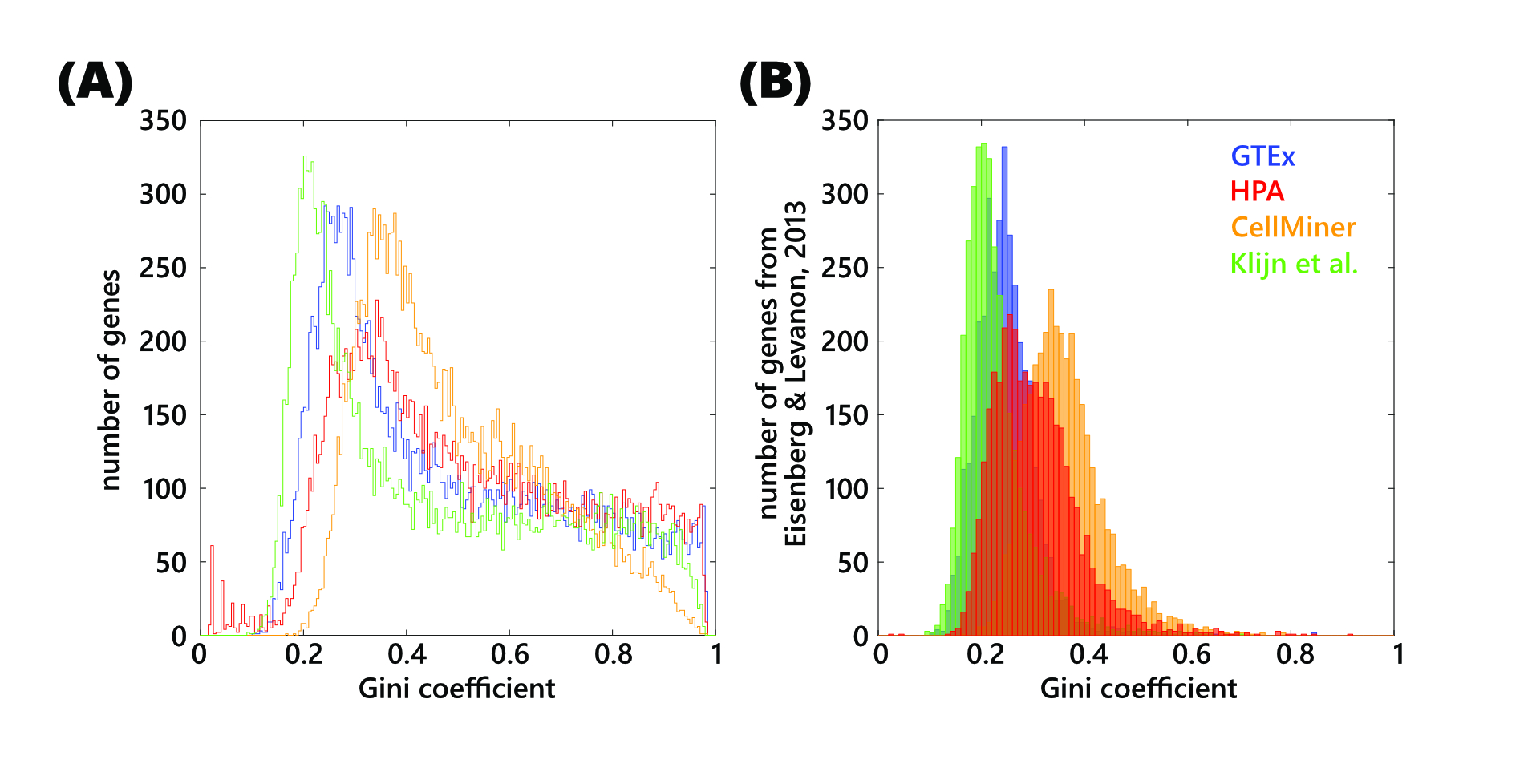

Supplement: S1 Fig — (A) The distribution of the Gini coefficients of all the genes for each of the datasets. (B) The distribution of the Gini coefficients of only the list of genes published by Eisenberg & Levanon. (TIF) [file pcbi.1010295.s002.tif]

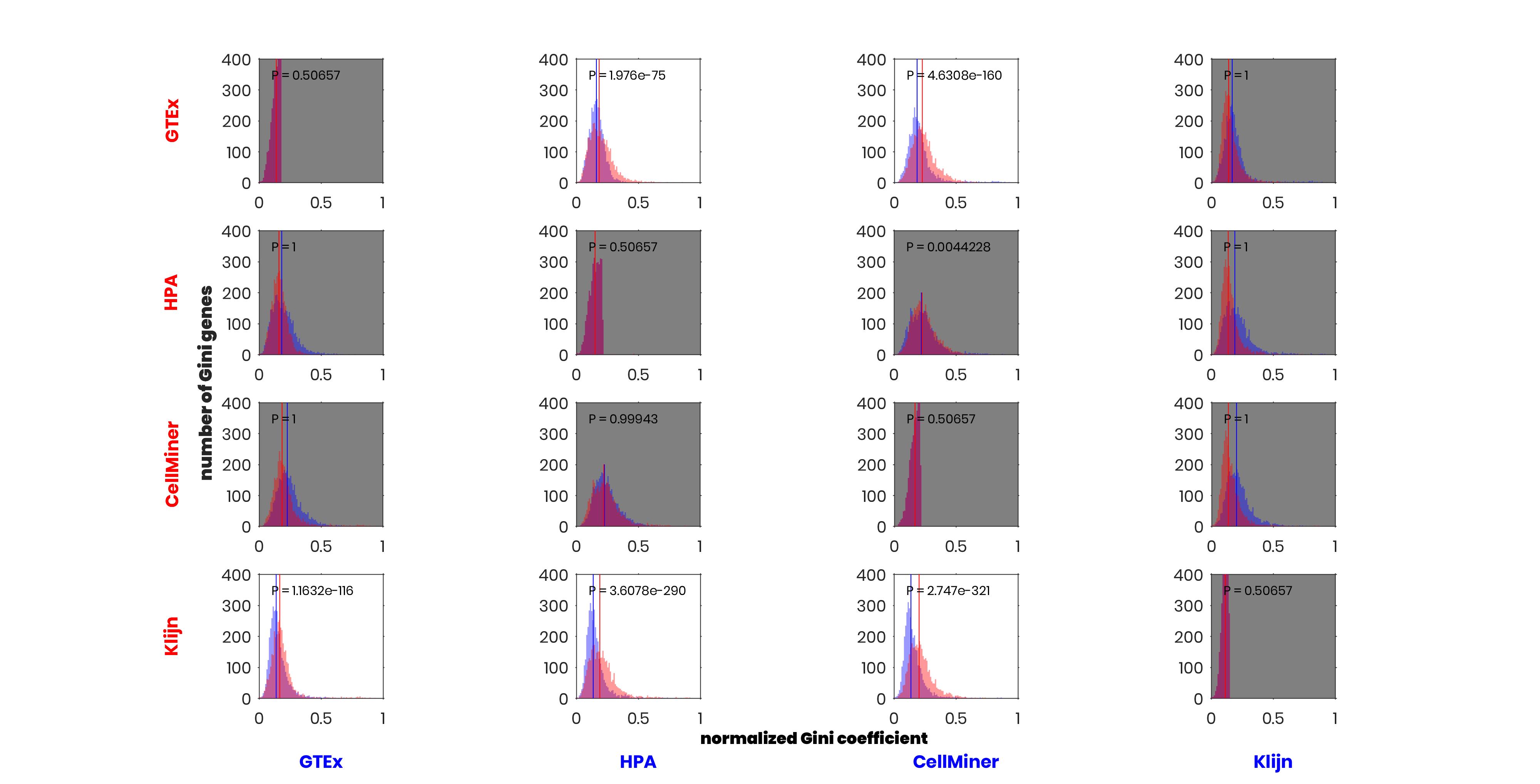

Supplement: S2 Fig — Gini genes identified using one dataset had lower Gini coefficient in the other dataset. The grey background plots are those where the P-value calculated suing Wilcoxon rank sum test was not significant (p <0.00001). (TIF) [file pcbi.1010295.s003.tif]

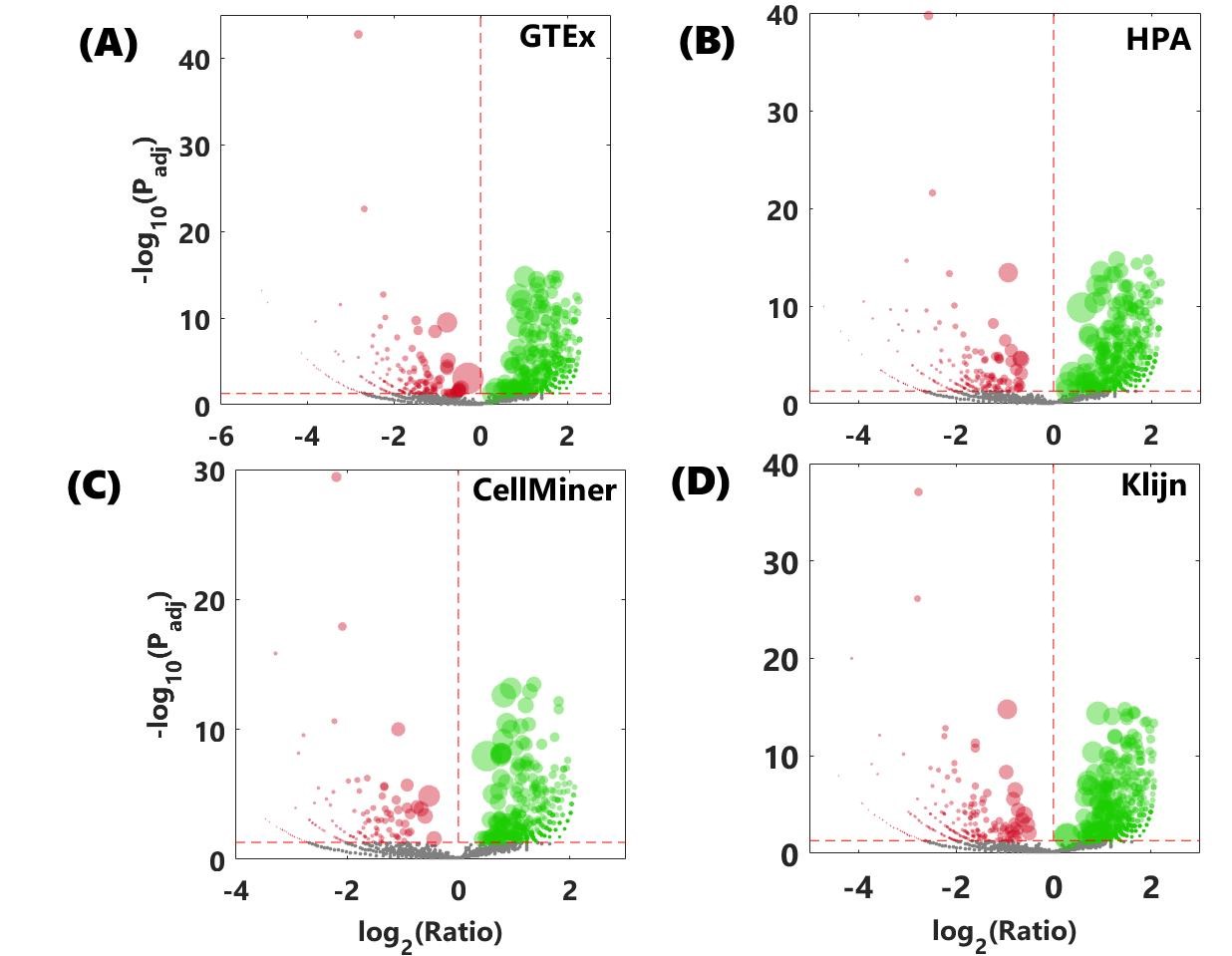

Supplement: S3 Fig — Volcano plots for GO term enrichment analysis for GTEx (A), HPA (B), CellMiner (C), and Klijn et al [37]. (D). The colors green, red, and grey indicate the GO terms which were over-represented, under-represented, and not enriched. The size of the bubble indicates number of genes belonging to a GO term. The x-axis represents the ratio of number of hits in the list of Gini genes to those achieved at random. (TIF) [file pcbi.1010295.s004.tif]

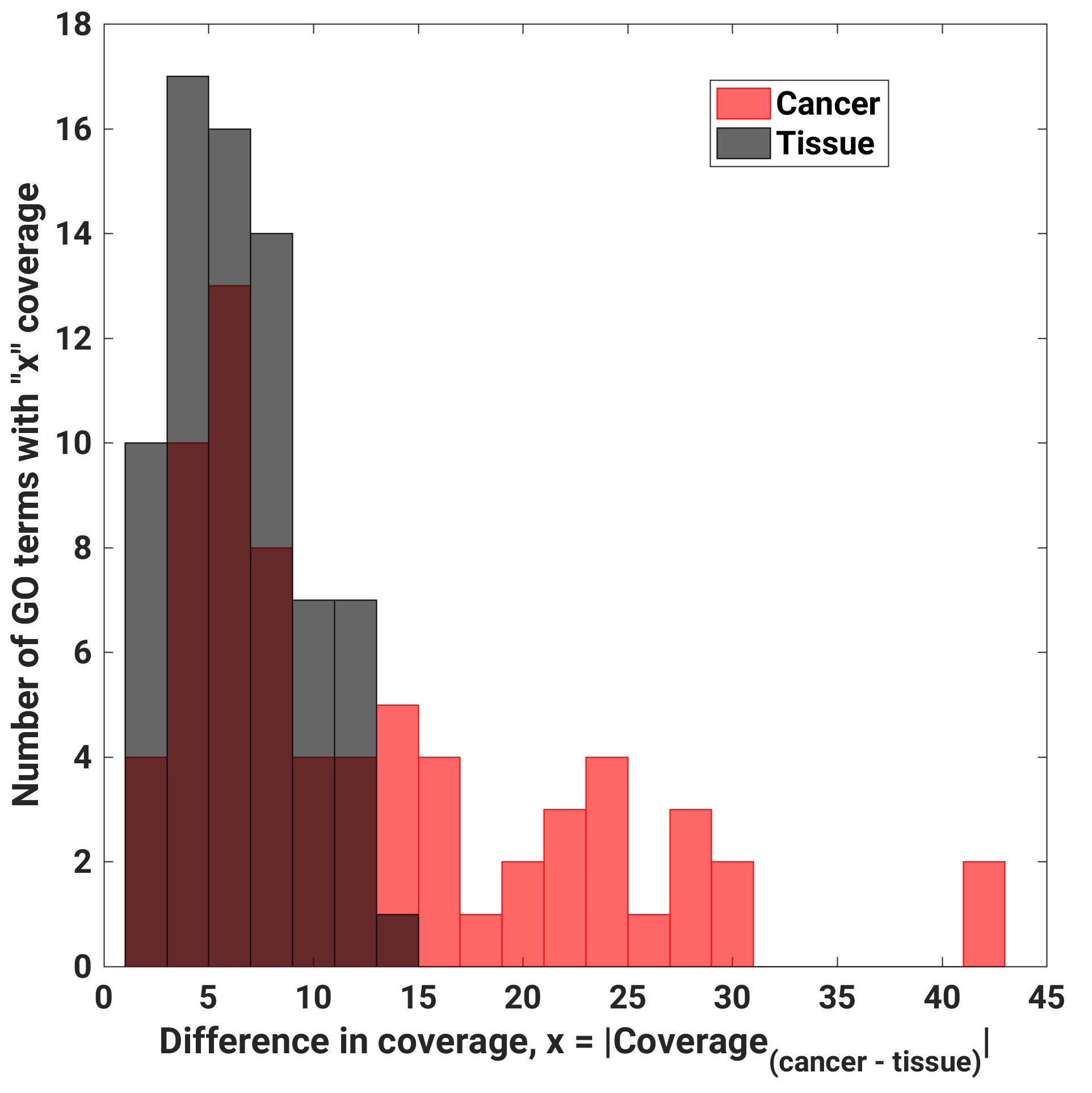

Supplement: S4 Fig — Red bars for cancer cells and Black bars are for tissues. The full data used to prepare this plot is in S1 Table. Included in this plot are 70 GO terms which are only enriched in cancer datasets but in neither of the tissue datasets, and 77 GO terms which are only enriched in tissue datasets but in neither of the cancer datasets. (TIF) [file pcbi.1010295.s005.tif]

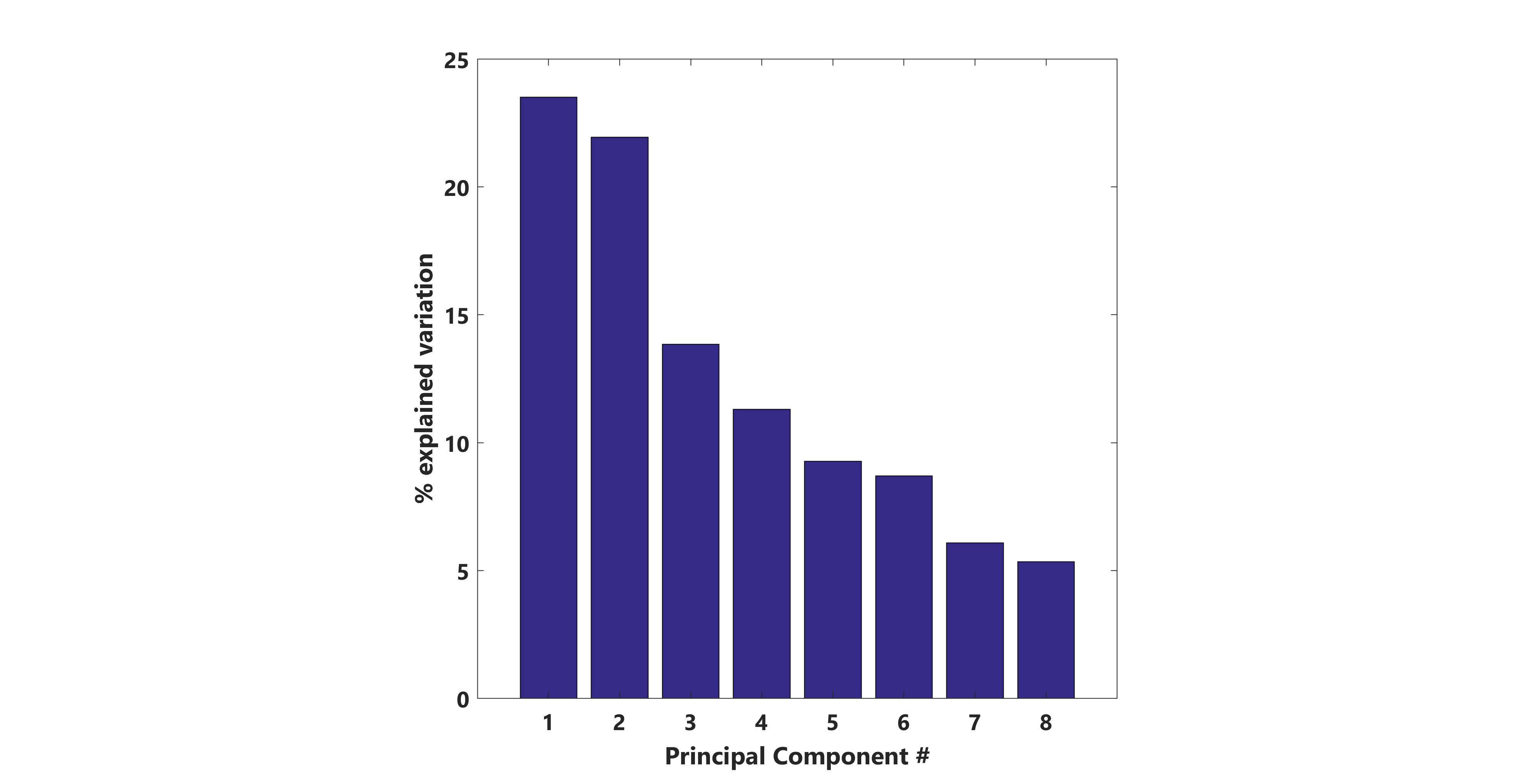

Supplement: S5 Fig — The PCA was performed using 1:1 ortholog Gini coefficients which were calculated using transcriptomes in Brawand et al. [35] to cluster organisms. (TIF) [file pcbi.1010295.s006.tif]

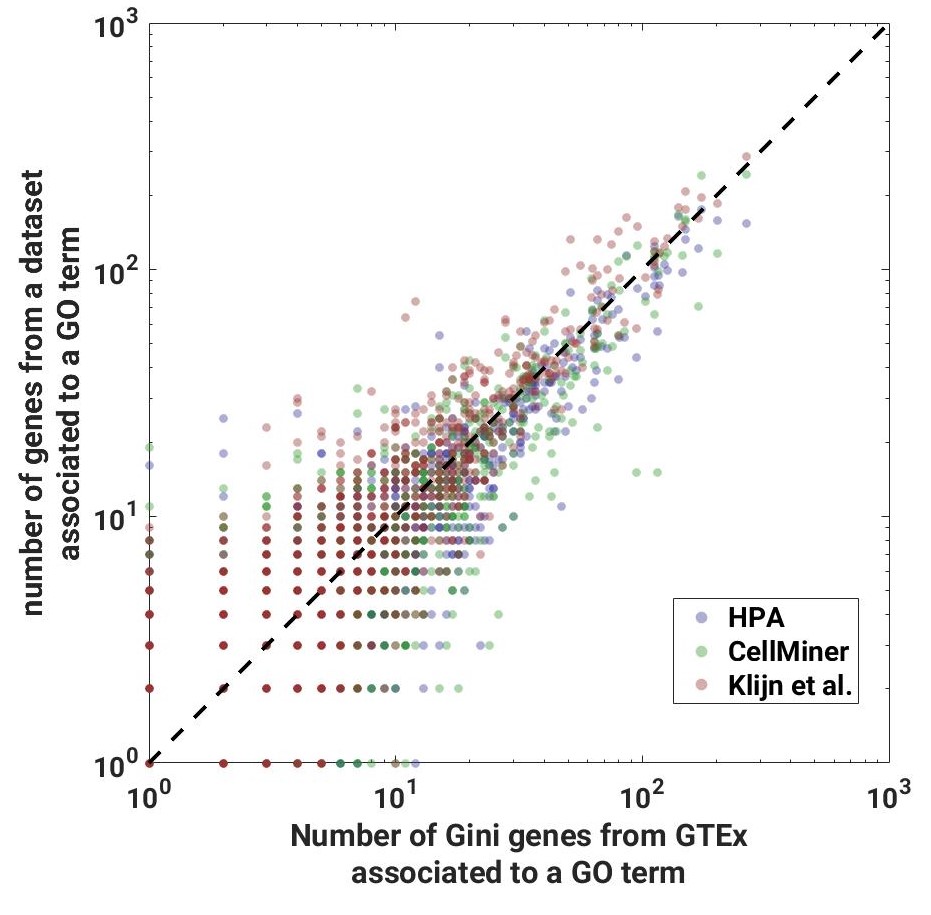

Supplement: S6 Fig — HPA/GTEx comparison represented in blue, CellMiner/GTEx in green, and Klijn [37]/GTEx in red. (TIF) [file pcbi.1010295.s007.tif]

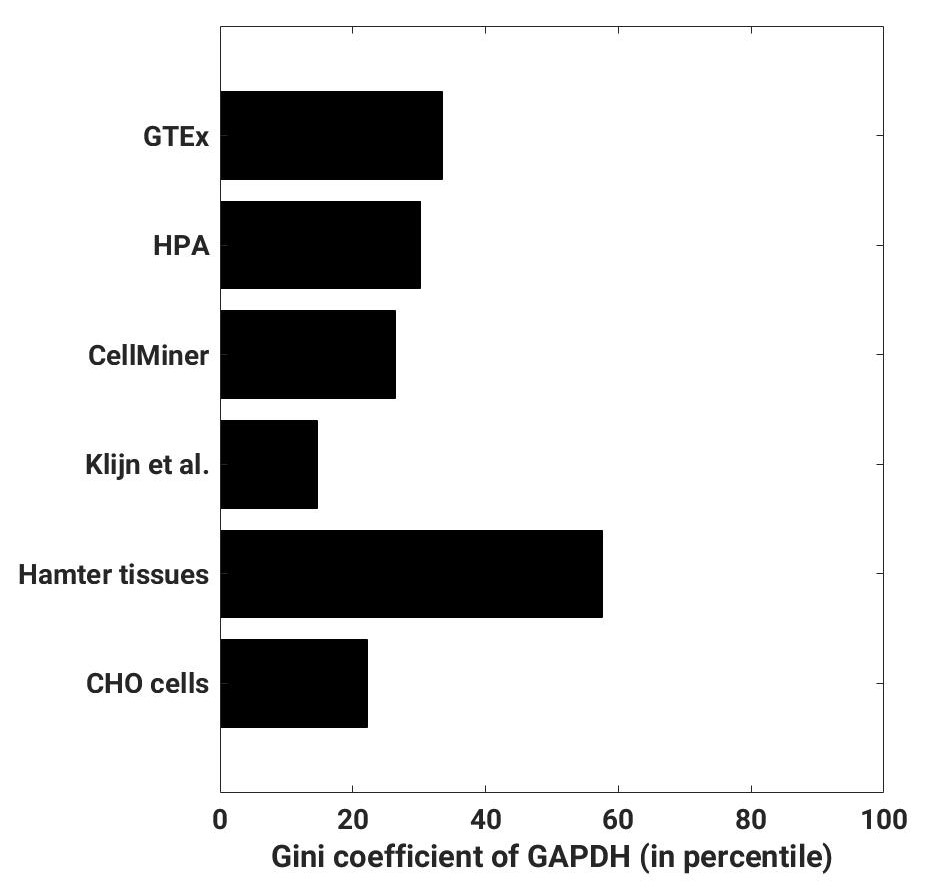

Supplement: S7 Fig — Gini coefficients were converted to percentiles (x-axis) using each of the datasets (y-axis). GAPDH has high Gini coefficient in most of the datasets. (TIF) [file pcbi.1010295.s008.tif]

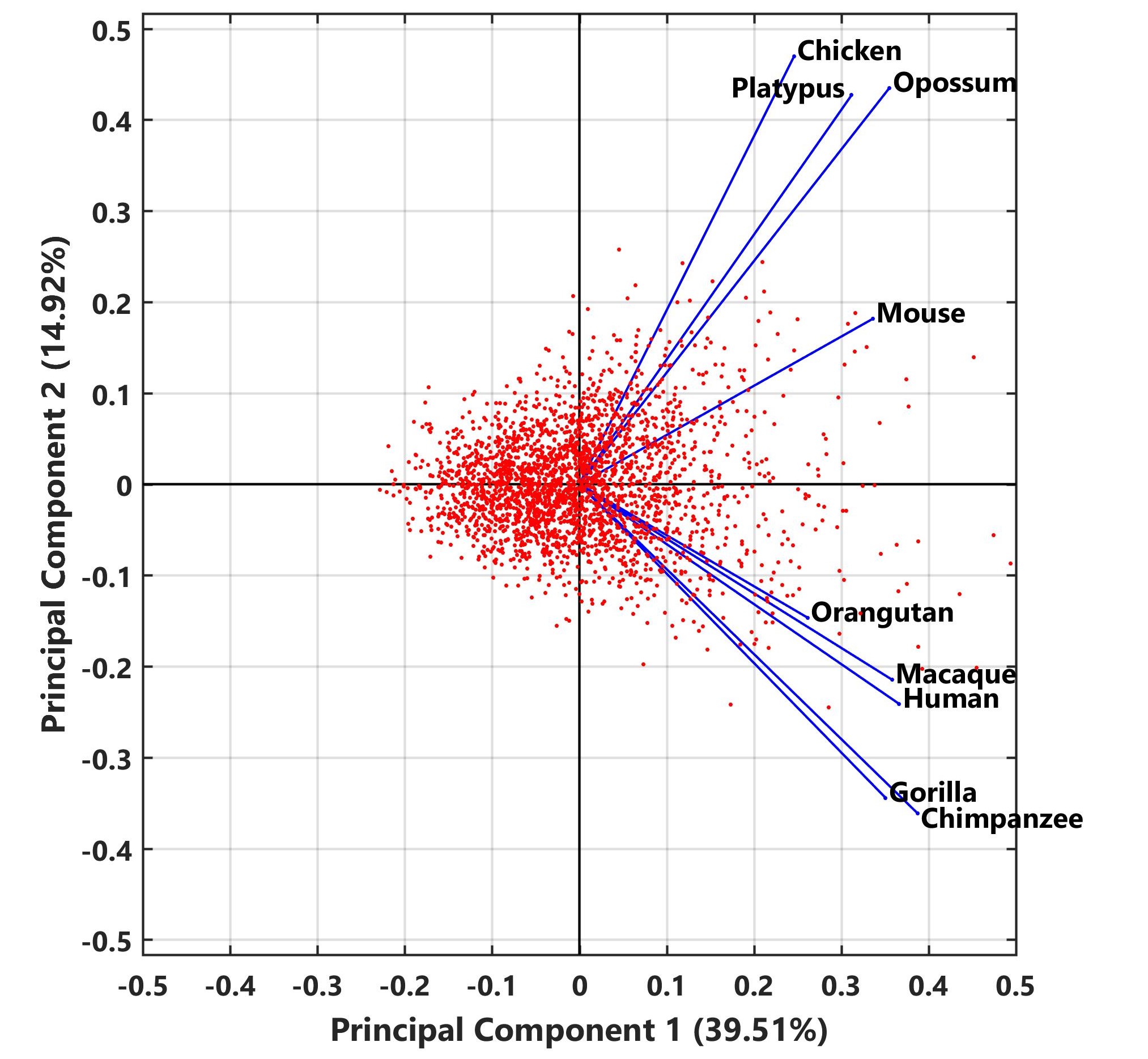

Supplement: S8 Fig — The first principal component which captures majority of explained variation does not explain Gini values in either of the organisms as all of them lie to the right-hand side of the plot. (TIF) [file pcbi.1010295.s009.tif]

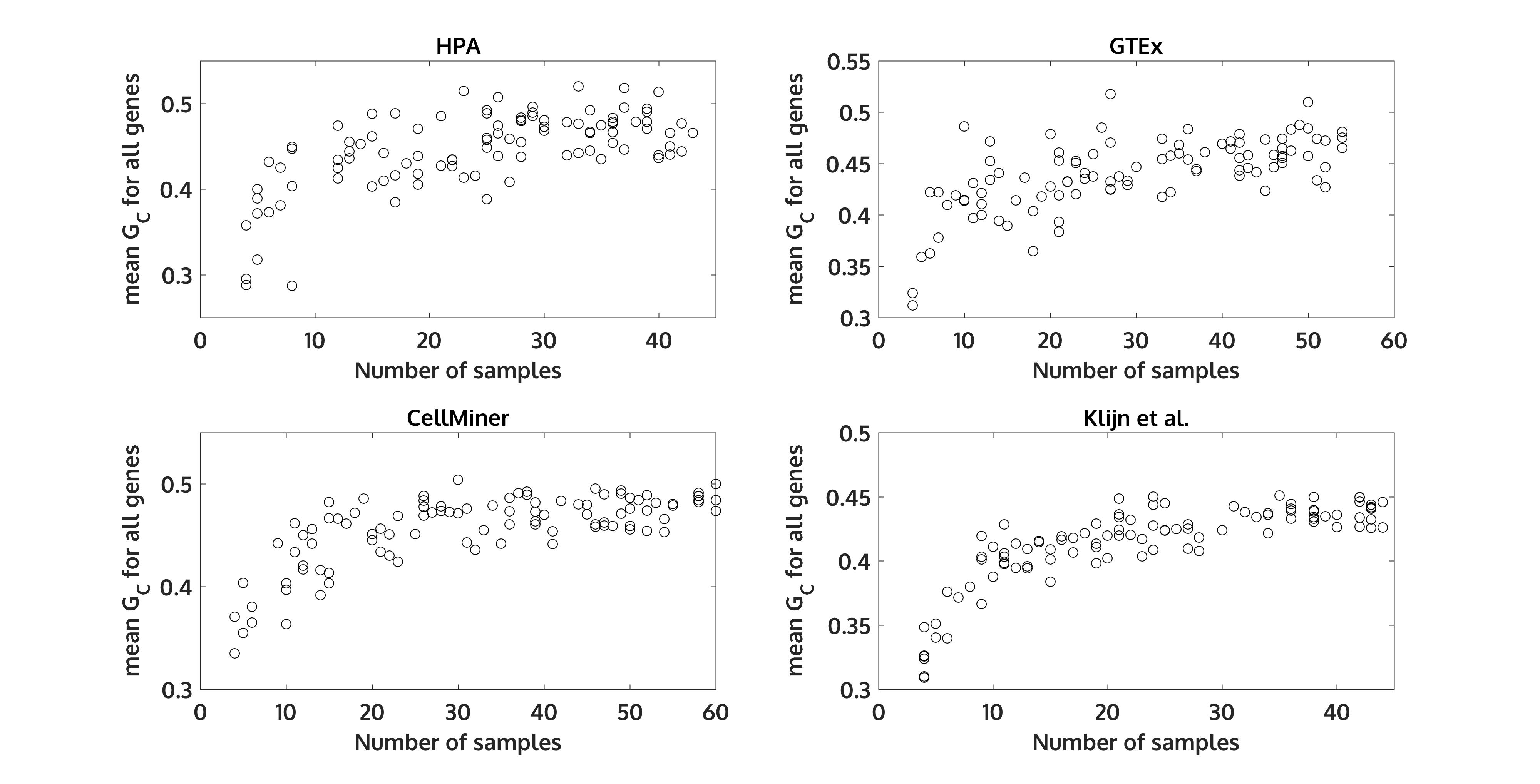

Supplement: S9 Fig — Each dot represents the mean GC value of all the genes. The figure was generating by randomly selecting arbitrary number of samples (less than the total number of samples) 100 times. (TIF) [file pcbi.1010295.s010.tif]

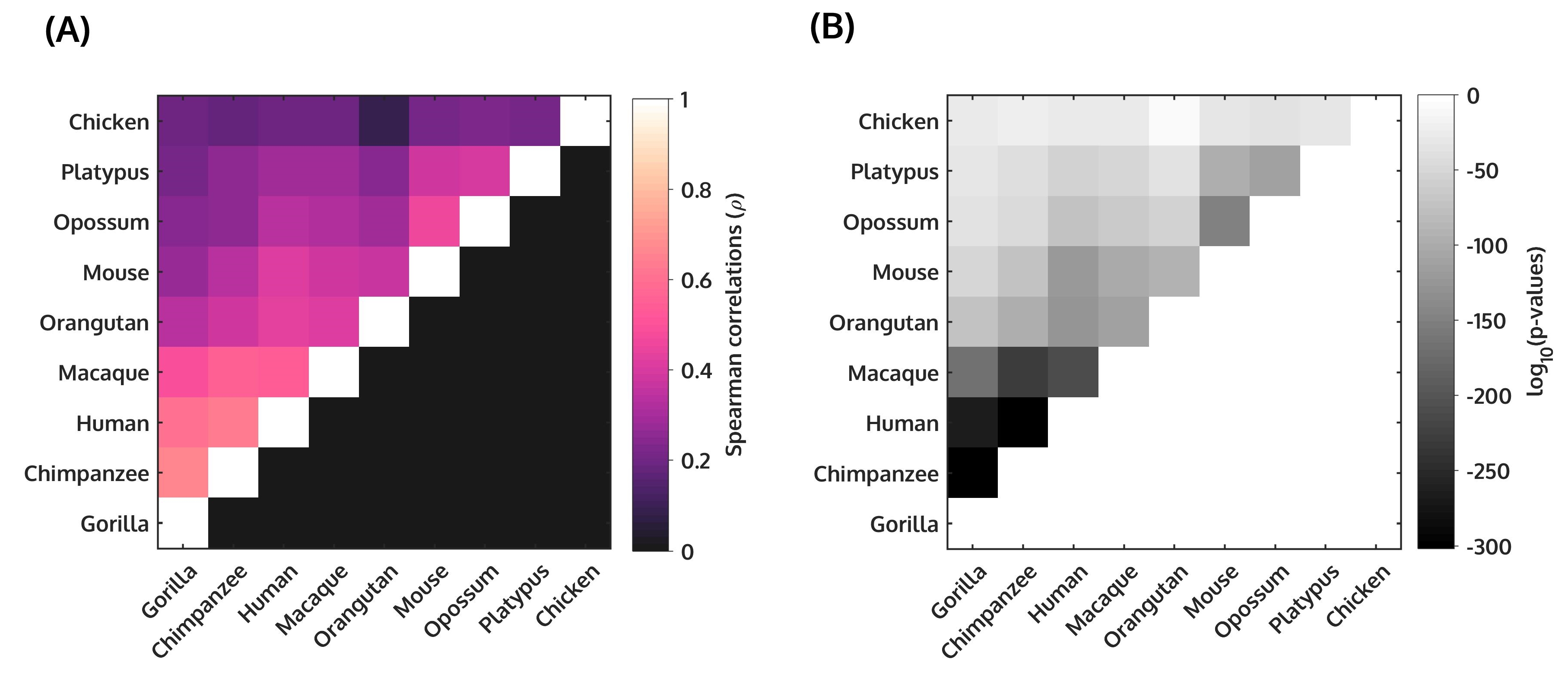

Supplement: S10 Fig — (A) Spearman correlations of Gini coefficients of Gini genes (bottom 20th percentile of GC in at least one organism) identified using organism-specific transcriptomes capture cluster containing primates. (TIF) [file pcbi.1010295.s011.tif]
