# Supplementary material for: What are housekeeping genes?
Source: PLoS Comput Biol. 2022 Jul 13;18(7):e1010295. doi: 10.1371/journal.pcbi.1010295 (PMC9312424; doi:10.1371/journal.pcbi.1010295)
Supplement: Attachment — Submitted filename: Reviews_revFinal.docx [file pcbi.1010295.s018.docx]

# Summary

We thank all the reviewers for their helpful suggestions. Their comments have led to the following changes:

1. Significant addition to the discussion section.
2. Addition of statistics where necessary.
3. Clarification of G_C_ thresholds
4. Addition of new supplementary information.

Please see our responses throughout this document. The reviewer concerns are in bold and our responses are in normal.

# Reviewer 1

**Reviewer #1: The manuscript "What are housekeeping genes?" by Joshi et al. revisits the definition of housekeeping genes. The authors summarize commonly-used criteria for housekeeping gene properties and argue that the inter-relationship between different criteria has not yet been systematically assessed. A quantitative measure of Expression Stability is proposed as a continuous proxy for 'housekeepingness' and its relation to the gene function, essentiality and conservation across taxonomic groups is analyzed on the basis of publicly available data resources.
The manuscript is concise and very-well written and structured. Plots support the main results and statements. I have a few comments/suggestions:**

**- Lines 103-104: I understand that the authors aimed to have the same number of candidate housekeeping genes (n = 3688) between the Eisenberg & Levanon benchmark gene set and the different test data sets (HPA, GTEx, CellMiner, Klijn). Yet, I would expect that the coverage of Gini value-based selection of housekeeping genes in the test data sets with the benchmark is, in part, dependent on the number of genes in each data set. E.g. What are the total gene numbers in each data set? The first paragraph in the results section states only sizes of combined data sets. A different approach to gene selection could be to select x% of genes with the lowest Gini values per data set and thereby better account for the different gene numbers in the different data sets. Perhaps the authors could elaborate on the motivation behind their gene selection approach and contrast their choice with alternative selection methods.**

In this study we chose to include the 3688 genes with lowest Gini values for a variety of reasons: (1) The gini coefficients of genes which are missing in either one of the datasets are not proportionally distributed (Fig F1). However, the likelihood of a gene with lower Gini coefficient not being included in the comparison was lower. (2) Without the necessary data available, there is no way to remove batch effects when comparing human tissue datasets (HPA versus GTeX), cancer datasets (Klijn et al. versus CellMiner), or accurately attribute expression levels to changes in conditions when comparing human tissue versus cancer datasets to bring them at par. (3) There isn’t a way to biologically justify the use of certain value other than to make comparisons possible. (4) By selecting the x% of genes with lower Gini values may not account for proper comparison with the 3688 genes in Eisenberg et al. These genes were determined using a different technology (microarray) altogether. However, we do employ picking the x% of genes with lowest Gini values when using the data from Brawand et al. 2011 data because these were generated as part of the same experiment. Therefore, in that case, it is safe to assume that all the differences are attributed to organismal differences and not batch effects. We have discussed these arguments in the discussion section. We have addressed these in discussion.

**- One of the mentioned criteria for 'housekeepingness' is that these genes are consistently expressed across tissues and conditions (i.e. Expression Stability). If I understood it correctly, this criterion is directly represented in the calculated Gini coefficient with low values indicating similar expression rates across treatments/tissues. Thus, the interpretation of low Gini coefficients to be associated with consistent expression can only be made for the conditions and tissues tested, while the possibility exists that inferred 'housekeeping' genes could display differential expression in a yet untested condition or e.g. not yet investigated organism developmental stage. This risk/limitation still exists with the housekeeping gene definition proposed by the authors. I invite the authors to elaborate on this common issue in housekeeping gene definition and the limitations of quantitative housekeeping definition that is proposed.**

We have clarified this with the additional sentence: “Therefore, there will remain a possibility of encountering genes that weren’t previously considered as housekeeping.”

We have proposed using “housekeeping functions” rather than “housekeeping genes”, see lines 307-314. We further added additional sentences: “Further, the housekeeping genes are often discussed in the context of organisms and not the environmental condition of the organism. For instance, when defining organismal housekeeping genes, the stability of samples isn’t accounted for or discussed. Our GO term analyses clearly showed the significant differences in housekeeping genes in cancer cells versus those for tissues (Fig. S4). Therefore, there will remain a possibility of encountering genes that weren’t previously considered as housekeeping.”

***minor comments*
- Lines 66-68: Since I'd expert a broad readership for this paper, it would be great to provide a brief description why it is important to identify minimal gene sets supporting life.**

A minimal list of genes which can support life can be important to understand the most evolutionarily resilient genes whose functions continue to persevere after millions of years of evolution. It has also been argued that minimal gene sets are dependent on the taxa and environment of the organisms. The existence of genes which continue to be part of minimal lists in many different organisms will highlight the key genes critical to support sustainable healthy life on earth. We have added discussion on this.

**- Lines 103-104: Also, the sentence should ideally be split into two; the second starting with "This gene set allowed [...]"**

Done

**- Line 112: "had low G C values". This is a bit vague/imprecise in my opinion. I would suggest to express this statement more quantitatively, e.g. in terms of median Gc values and their position in the Gc value distribution.**

We have added the 90^th^ percentile ratios of Gc of Eisenberg and Levanon et al. housekeeping genes to the same of the entire dataset. We have added the following the main paper “…values in each of the datasets compared to the 90^th^ percentile G_C_ of these datasets (90^th^ percentile ratios: 0.49 for HPA, 0.37 for GTEx, 0.61 for CellMiner, and 0.37 for Klijn et al.”

**- The gene coefficient symbol "G_C" is sometimes written with C in the subscript, sometimes with a lower case "c". This should be unified.**

We have fixed these.

# Reviewer 2

**Reviewer #2: The definition and conception of housekeeping genes were ambiguous for decades. This study presented a quantitative approach to define the housekeeping genes from four aspects, including expression stability, cellular maintenance, essentiality and conservation. Expression stability was scored by Gini coefficient (Gc); cellular maintenance was detected by GO term; essentiality was identified through the influence of development levels; conservation was defined by finding the consensus signature through different organisms. The study is timely and may be interest for the readers of the journal.

I have number of comments to improve the presentation of the paper before its publication.

Major comments**

**1. The potential link among the four terms (expression stability, cellular maintenance, essentiality and conservation) were still unclear, and some data applied in the study may not fit. I suggest the authors discuss this in the manuscript.**

Yes, in part this is the motivation for this study. While expression stability and essentiality are extensively discussed till date. There is little systemic literature on list of cellular maintenance genes or conserved genes. And yet these terms are often used in communication of scientific literature. In this article, we have presented a list of genes which may be conserved in a variety of organisms. This data is presented in Supplementary Tables. For example, GO id enrichment is presented in Table S1 and Table S2, and Gini genes (17.5 percentile genes with lowest Gini coefficient are suggested as housekeeping genes) are presented for all the data used in the article in Table S3. In revised supplementary information, we have also added Gini genes which were presented in each of the organisms from Brawand et al. We have added this discussion in the revised manuscript.

**2. The application of cancer data may confuse the readers. It had been reported that housekeeping genes could have different performance in cancer compared with normal tissues, and the results in this study also support the conclusion. For example, the low overlap fraction of low Gc genes and housekeeping genes, the enriched GO terms of cancer data had low connection with basic cellular maintenance, and so on. Please discuss this in the manuscript.**

We have added this in the revised manuscript.

**3. The application of whole-body single cell RNA-seq data of worm may confuse the reader. Compared with bulk data, the major advantage of scRNA-seq is the expression data at cell type level, but the advantage in the usage of cell type data has not been clearly shown in the paper. The disadvantage of scRNA-seq technique that it has low sensitivity to low expression genes, may have an adverse effect while calculate Gc. Additionally, the sc data was generated from an individual worm larval stage, which can cause unstable results. The authors may discuss this in the manuscript and revise it accordingly.**

We agree with the reviewer and we have added discussion of these caveats in the revised manuscript.

**4. Some results were not clearly described. In figure3, the relationship between the expression level and Gc lack the support of statistics, for example, spearman correlation. In figure4D, the direct evidence of the overlapping between essential genes and low Gc genes was required, which can be showed by Jaccard index, hypergeometric distribution, Fisher exact test, and so on, instead of the number of genes of enriched GO terms. In figure5B and figure 5E, p values were required to show the significance of spearman correlation coefficient.**

For Figure 3, the following sentence was added to the figure caption: “The Spearman correlations between Gini coefficients and median expression values were -0.6545 (p = 0) and -0.7244 (p = 0) for HPA and GTEx, respectively.” For Figure, considering that CRISPR data and transcriptomics data are coming from different experiments, it is hard to do a Jaccard index analysis. Therefore, we provide comparison of medians using Wilcoxon p-values when comparing G_C_ of essentials with that of housekeeping genes and the total dataset. The larger p-values for comparison between essential genes and housekeeping genes indicate that these lists have similar medians. These were also supported by Kolmogorov-Smirnov test suggesting whether the G_C_ values are coming from similar cumulative distribution functions. Together, these suggests that housekeeping genes and essential genes lists are similar. The p-values are now reported in the Figure 4 captions. Due to the lack of systemically defined housekeeping genes for C. elegans and CHO cells. A similar comparison or statistic could not be drawn for these organisms (Figure 4C and Figure 4E). Therefore, we had initially provided the comparison of the correlation among the GO-terms. However, for Figure 4E, we do provide a sign-test (Table 1) comparing G_C_ values of essential genes in different groups is lower than that of all the genes together.

**5. This study processed transcriptomics datasets from different data sources such as HPA and GTEx. How many tissues were analysed by the study? Please describe the details in the method section. In addition, it would be interesting to add discussion about the median expression (in Figure 3A &B) of housekeeping gene (3688 genes published by Eisenberg and Levanon across tissues.**

We have addressed this with the following in figure 3: “Specifically, the median expression value within the 25^th^, 50^th^ and 75^th^ percentiles of the HPA dataset, were 12.2, 22.1, and 41.5 transcripts per million (TPM), respectively; whereas within the GTEx dataset, the 25^th^, 50^th^ and 75^th^ percentile values were 14.2, 24.7, and 46.4 TPM, respectively.”

**6. The benchmark gene selection. The published paper from Eisenberg and Levanon found 3804 housekeeping genes, and in the presented manuscript, only 3688 genes were adopted. There should be a description about the criteria of benchmark gene selection. The authors may discuss this in the manuscript and revise it accordingly.**

Yes, this discrepancy arises because some of the genes could not mapped when gene nomenclature was changed. We used Biomart to change the nomenclature from gene names to Entrez Gene ID. We have added an extra row in Table 2 in the Methods section.

**7. Why cancer expression profiles (NCI-60) were included when screening housekeeping genes? The results here showed housekeeping gene/GO term overlapping between tissue and cancer were not very consistent, so will this shrink or enlarge the possible range of candidate genes in functional manner? Considering some cancer types have poor 3-year survival rate, which could lead to unstable cell condition when sampling for RNA-seq, so it necessary to also include expression profiles of chronic diseases.**

The definition of housekeeping genes does not require the stability of conditions. The cancer expression profiles were useful because it is a large dataset with expression profiles coming from the same experiment. As we have noted and discussed that it was interesting to find differences/inconsistencies between housekeeping genes identified using cancer and human datasets. In the discussion section, we have added the following: “Further, the housekeeping genes are often discussed in the context of organisms and not the environmental condition of the organism. For instance, when defining organismal housekeeping genes, the stability of samples isn’t accounted for or discussed. Our GO term analyses clearly showed the significant differences in housekeeping genes in cancer cells versus those for tissues (Fig. S4).”

**8. GO terms in cancers showed different emphasized aspect differ from normal tissues, which concentrating in cell cycle processes. This is conflicting with the definition of housekeeping genes.**

We have discussed in the discussion section. We have added discussion on this.

**9. The authors suggested housekeeping functions, rather than housekeeping genes, may be suitable in describing the organism status. It’s more convincing if there is comparison between the housekeeping functions and genes when it come to the correlation among different tissues/cell lines.**

In the biological hierarchy of cells, genes are a precursor to functions. In our argument, housekeeping genes maybe an overly-specific to describe the concept of which cellular processes are important for cell survival. By describing housekeeping functions, the slighter nuances in expression of individual genes could be minimized; thus, enriching global understanding of important cell survival. We do present a list of GO ids associated with housekeeping genes in tissue, cancer, and multi-organism datasets in Table S1 and S2.

**Minor comments**
**1. As described in the abstract, except human data, data from other 12 organisms were included. However, only 11 species can be found in the main text. The results of GO terms containing p-values are expected.**

We have fixed 12 to 11. We have added the p-values now.

**2. In figure4, the arrangement of plots should follow the same order.**

We have fixed this.

**3. Authors mentioned that data without TPM value were converted from read per base, we suggest to provide the detailed calculation.**

The formula for the calculation has been added to Table 2. Further, all of the transcriptomics data is available on the Github page for this tool. The link to the Github can be found in the main manuscript.

**4. Figure 5B, the legend shows spearman correlation but the caption is about Jaccard similarity.**

Thank you for catching that. We used spearman in the analyses presented in the figure. We have made the edits in the figure caption.

**5. The authors should state clearly which statistic method they used in correlation calculation part of Figure 2B.**

We have used spearman and has been added to the figure caption.

**6. The criteria of “low Gc value” should be stated clearly.** Done.

# Reviewer 3

Reviewer #3: What are housekeeping genes?
PCOMPBIOL-D-22-00176
Review

**Summary of the research and overall impression
The Authors discuss the concept of housekeeping genes analyzing four properties: stable expression across cell types and conditions, involvement in basic cellular maintenance functions, essentiality, conservation across evolution.
The Authors use the GC approach to identify stably expressed genes (Gini genes) across human tissues and cell lines, and across cell types of lower organisms studied using single-cell transcriptomics. Then they compare the properties of stability (GC coefficient) to the property of essentiality, as defined by in vitro or in vivo functional genomics studies.
GC values are highly correlated across human datasets, supporting the existence of a subset of genes stably expressed across cell types and conditions.
Further, genes that are stably expressed and essential are associated with GO terms enriched in basic cellular functions, and also are more likely to be conserved.
Although the four properties informally used to define housekeeping genes are more likely than not to associate with each other, they are not strictly linked. In conclusion the results show the need for organism-specific tools and models.

The topic is of interest for the scientific community and the work presents a paradigm that can be broadly and usefully applied in the field of quantitative gene expression analyses.

Major issues
In general, the methods are adequate, but the manuscript needs to improve in clarity and readability. Please find below my suggestion.**

**Introduction**
**The first part of the Introduction is confounding. You should state a general definition of HF (a large class of genes that are constitutively expressed, subjected to low levels of regulation in different conditions and perform biological actions that are fundamental for the basic functions of the cell CIT Eisenberg E and Levanon EY: Human housekeeping genes, revisited. Trends Genet 29: 569-574, 2013.) Then, my suggestion is to follow the general idea of the four criteria: stability, maintenance, essentiality, and conservation, always in the same order along the manuscript. If the order will be maintained, the manuscript will improve in readability.
In particular, in the Introduction I think the state referring to normal conditions (“they can be defined as genes stably expressed in all cells of an organism under normal conditions irrespective of tissue type, developmental stage, cell cycle state, or external signal, or as markers of an organism’s healthy biological state is confounding”) is confounding. My suggestion is to focus on the four properties and to add a paragraph to discuss the idea the normal and pathological condition show different patterns of gene expression and also different HK genes.**

We have made changes to the Introduction and addressed some of these concerns in the discussion section.

**Results**
**The Results session is well organized because it follows the description of the four properties, but since the Authors performed analyses on human tissues, Human NCI-60 cancer cell lines, other organisms you should better specify the results obtained in each tissues/cells/organism in each paragraph of the Result session.**
Lines 187-244 Session results, Paragraph “Housekeeping genes are essential”:
**I suggest starting the paragraph with a definition of “essentiality”.** We have added this now.
**Is it possible to obtain data related to essentiality in human normal cell? Why did you focus on essentiality analysis only in cancer cell lines and C. elegans? You should clarify that you have performed analyses only for those two cell types and add a paragraph in the discussion.**

We have added in discussion section caveats with analyses in C. elegans. The data for essentiality is healthy human tissues isn’t available, in part due to ethical concerns. Further, the phenotypic diversity in essentiality of normal human tissues is hard to get. In this study we used, GTEx and HPA data. The essentiality for these tissue types does not exist. In this study, we analyzed essentiality for three cell types: cancer and CHO cells using CRISPR screens, and C. elegans using whole-body RNAi screens. As for as essentiality for diverse healthy human tissues are concerned, the data is hard to obtain for numerous reasons among which are ethical considerations, non-invasive measuring techniques, lack of knowledge on how to grow healthy human tissues, etc. However, this comparison was easier to show for C. elegans. However, the analyses presented here for C. elegans, throw light on whole-body essentiality. Indeed, this is not the same as measuring tissue essentialities and are hard to do for multi-cellular organisms and yet preserve the tissue-specific nature.

Discussion
**Lines 286-299 You should add references: not only the references already used in the Introduction, but you should also add references related to previous research (e.g. Eisenberg E and Levanon EY: Human housekeeping genes, revisited. Trends Genet 29: 569-574, 2013; Casadei R, Pelleri MC, Vitale L, Facchin F, Lenzi L, Canaider S, Strippoli P and Frabetti F: Identification of housekeeping genes suitable for gene expression analysis in the zebrafish. Gene Expr Patterns 11: 271-276, 2011; Zhang Y, Li D and Sun B: Do housekeeping genes exist? PLoS One 10: e0123691, 2015; Caracausi M et al. Systematic identification of human housekeeping genes possibly useful as references in gene expression studies, MOLECULAR MEDICINE REPORTS, 2017).**

We have added more references.

**As said before, I suggest the adding of a paragraph discussing the results with a specific reference to different cell types/organisms and a paragraph discussing the choice of analyzing only cancer cell lines and C. elegans in the context of the property essentiality.** The advantage of cancer cell lines are that these can be grown in the lab. This is in stark contrast to getting tissue-specific essentiality data for any of the multi-tissue orgnaisms. We attempted to do this for C. elegans. However, the essentiality data is for whole-body. We have discussed this in the discussion section.

Minor issues
Line 174
**“could be become targets”: please cancel “be”.**

Done.
